# Bacteriocin production of the probiotic *Lactobacillus acidophilus* KS400

**DOI:** 10.1186/s13568-018-0679-z

**Published:** 2018-09-27

**Authors:** C. Gaspar, G. G. Donders, R. Palmeira-de-Oliveira, J. A. Queiroz, C. Tomaz, J. Martinez-de-Oliveira, A. Palmeira-de-Oliveira

**Affiliations:** 1 Labfit-HPRD: Health Products Research and Development, Lda, Covilhã, Portugal; 2 CICS-UBI: Health Sciences Research Centre, Covilhã, Portugal; 3 Antwerp University Hospital, Antwerp, Belgium; 4 Femicare vzw clinincal Research for Women, Tienen, Belgium; 5 Child and Women’s Health Department, Centro Hospitalar Cova da Beira EPE, Covilhã, Portugal

**Keywords:** *Lactobacillus acidophilus* KS400, Vaginal probiotics, Vaginal lactobacilli, Bacteriocins, Vaginal infections

## Abstract

In the last years, the use of probiotics, including *Lactobacillus* species, has received much attention to prevent and treat vaginal disorders. These species have been described as having the ability to colonize the epithelial surface and produce antimicrobial metabolites that are able to control the remaining vaginal microflora. This study aimed to identify and characterize, for the first time, a bacteriocin natively produced by *Lactobacillus acidophilus* KS400 (probiotic strain from Gynoflor^®^-Medinova AG, Switzerland) and its antimicrobial activity against relevant urogenital pathogens. After organic acids and hydrogen peroxide neutralization in the fermented *Lactobacillus acidophilus* KS400 culture medium, bacteriocin activity was tested against the indicator microorganism *Lactobacillus delbrueckii* ATCC9649. The fermentation of *Lactobacillus acidophilus* KS400 for bacteriocin production was carried out in batch mode, and its antimicrobial activity, optical density and pH were monitored. After production and extraction, the bacteriocin molecular weight was estimated by electrophoresis and tested against vaginal pathogenic microorganisms. As described for other bacteriocins, batch fermentation profiles indicated that bacteriocin production occurs during the exponential growth phase of the lactobacilli, and declines during their stationary growth phase. The molecular weight of the bacteriocin is approximately 7.5 kDa. The bacteriocin containing protein extract was shown to inhibit the growth of *Gardnerella vaginalis*, *Streptococcus agalactiae*, *Pseudomonas aeruginosa* and the indicator strain *Lactobacillus delbrueckii* ATCC9649. We conclude that *L. acidophilus* KS400 produces bacteriocin with antimicrobial activity against relevant urogenital pathogens.

## Introduction

The term probiotic was derived from the Greek ‘*biotikos*’, meaning “for life” and refers to “live microorganisms which when administered in adequate amounts confer a health benefit on the host” (FAO 2006). In last three decades, the use of probiotics has received much attention as a treatment and prevention option for vaginal disorders (Barrons and Tassone 2008; Coudeyras et al. 2009; Homayouni et al. 2014). The equilibrium between the several microorganisms that compose the human vaginal microflora is important for the maintenance of its homeostasis, affecting directly the health status of woman (Gajer et al. 2012; Ravel et al. 2011). Among the prevalent microorganisms, the *Lactobacillus* species represent at least 70% (10^7^–10^8^ CFU (colony forming units)/g of vaginal fluid) of the bacteria identified in the reproductive system of healthy premenopausal women (Anukam et al. 2006; Ravel et al. 2011; Strus et al. 2005; Zhou et al. 2004). The most prevalent *Lactobacillus* spp. in the vagina are *L. crispatus*, *L. jensenii*, *L. gasseri* and *L. iners* (Pavlova et al. 2002; Vásquez et al. 2002; Zhou et al. 2010). In the vaginal epithelium, *Lactobacillus* induce probiosis in two stages: (1) colonizing the epithelial surface, competing for attachment sites and promote pathogen co-aggregation (competitive exclusion) (Coudeyras et al. 2009; Martín et al. 2012; Zárate and Nader‐Macias 2006); and (2) producing antimicrobial metabolite substances that are able to control the remaining vaginal microflora (microbial killing, inhibition) (Heng-Yi et al. 2008; Kaewsrichan et al. 2006; Voravuthikunchai et al. 2006). Within the different metabolites produced by *Lactobacillus*, organic acids (mainly lactic acid) and hydrogen peroxide are responsible for broad-spectrum microbial inhibitory effect (O’Hanlon et al. 2013; Tomás et al. 2003). On top of this, production of more specific antimicrobial proteins (bacteriocins) have been described (Aroutcheva et al. 2001; Pingitore et al. 2009; Turovskiy et al. 2009). Bacteriocins are molecules with proteinaceous nature which have bactericidal or bacteriostatic activity against closely related species (narrow spectrum) or across genera (broad spectrum) (Cotter et al. 2005; Klaenhammer 1993). Gram-positive bacteria, especially lactic acid bacteria (lactobacilli, lactococci and pediococci), produce these antimicrobial peptides and proteins in the ribosomes, such as lactacin B from *Lactobacillus acidophilus* (Barefoot and Klaenhammer 1983; Muriana and Klaenhammer 1991), plantaricin 423 from *Lactobacillus plantarum* (Van Reenen et al. 1998), pediocin ST18 from *Pediococcus pentosaceus* (Todorov and Dicks 2005), nisin Q from *Lactococcus lactis* (Zendo et al. 2003) and several others from other bacteria (Netz et al. 2002; Srionnual et al. 2007). Due all above characteristics, *Lactobacillus* species have been proposed as vaginal probiotic and used for the reestablishment of the vaginal environment balance, preventing growth of pathogens in the vagina (Kaewsrichan et al. 2006; Zárate and Nader‐Macias 2006). The safe use of *Lactobacillus* species as probiotic agents in human vagina dates back to 1915 (Newman 1915). In this in vitro study, we evaluated whether *Lactobacillus acidophilus* KS400 (LaKS400, Gynoflor^®^, Medinova AG, Switzerland) is a bacteriocin producer and its antimicrobial activity.

## Materials and methods

### Microorganism strains

The bacterial and fungal strains used in this study, including the investigated strain (*La*KS400), the bioactivity indicator strain (*Lactobacillus delbrueckii* ATCC9649) and urogenital pathogens selected to assess the protein antimicrobial activity are shown in Table 1. Bacterial and fungal strains suspended in adequate medium added with 25% of glycerol (VWR, Spain) and stored frozen at − 80 °C, until used.

**Table 1:** Bacterial and fungal strains used in this study

Microorganisms	Source	Growth condition (media, temperature and atmosphere)
*Lactobacillus acidophilus* KS400	Medinova	MRSB, 37 °C, 10% CO_2_
*Lactobacillus delbrueckii* ATCC9649^a^	ATCC collection	MRSB, 37 °C, 10% CO_2_
*Gardnerella vaginalis* 586876	Clinical	NYCIIIB, 37 °C, 10% CO_2_
*Gardnerella vaginalis* 563765	Clinical	NYCIIIB, 37 °C, 10% CO_2_
*Gardnerella vaginalis* 568799	Clinical	NYCIIIB, 37 °C, 10% CO_2_
*Staphylococcus aureus* ATCC6538	Collection	TSB, 37 °C, aerobic
*Streptococcus agalactiae* 181324	Clinical	BHIB, 37 °C, aerobic
*Streptococcus agalactiae* 179954	Clinical	BHIB, 37 °C, aerobic
*Pseudomonas aeruginosa* ATCC15442	Collection	TSB, 37 °C, aerobic
*Escherichia coli* ATCC8739	Collection	TSB, 37 °C, aerobic
*Candida albicans* ATCC10231	Collection	BHIB, 37 °C, aerobic

^a^Indicator microorganism

When gelose medium was required (for example, for the maintenance of microorganisms), bacteriological agar (VWR, Spain) was added to the liquid medium at the final concentration of 20 g/L. De Man, Rogosa and Sharpe (MRS) soft agar was prepared with 7.5 g/L bacteriological agar.

### Bacteriocin detection

LaKS400 was cultivated in 10 mL MRS broth (VWR, Spain) pH 6.5 for 24 h at 37 °C in the presence of 10% CO_2_. After incubation, the cell culture was heated at 70 °C for 30 min to assure inhibition of protease activity, then cooled at room temperature and centrifuged (4500 rpm for 15 min at 4 °C) with a benchtop centrifuge (Heraeus megafuge 8R centrifuge, Thermo-fisher Scientific, EUA). In order to eliminate the antimicrobial effect of organic acids, the pH of the supernatants was adjusted to 6.5 with 10 M NaOH solution. The inhibitory activity from hydrogen peroxide was eliminated by the addition of 5 mg/mL catalase from bovine liver (Sigma-Aldrich, EUA) followed by filtration through a 0.2 µm pore-size cellulose acetate (Fisher-Scientific, UK).

Bacteriocin detection was performed by using a modification of the antagonist well-diffusion method described by Tagg and McGiven (1971): briefly, 20 mL of MRS soft agar was inoculated with 200 µl of the indicator microorganism (*Lactobacillus delbrueckii* ATCC9649) in an overnight culture. Wells with 4 mm diameter were punched in agar plates and filled with 100 µl of cell-free culture supernatants. Phosphate-buffered saline (PBS) was used as negative control. The plates were maintained at room temperature for 3 h to allow for bacteriocin diffusion, followed by incubation at 37 °C in the presence of 10% CO_2_, during 24–48 h. The bacteriocin activity was determined by macroscopic observation of a clear inhibition zone on the agar.

### Bacteriocin production

To study the bacteriocin production kinetics, 500 mL of MRS broth at pH 6.5 were inoculated with *La*KS400 strain (1% v/v) and incubated at 37 °C in the presence of gaseous nitrogen without agitation. At every hour during fermentation, both bacterial cell density and the culture medium pH were measured. Every 2 h we proceeded to the evaluation of antimicrobial activity against the indicator strain (*Lactobacillus delbrueckii* ATCC9649) as described above.

For bacteriocin production, a 20 h old culture (10 mL) of *La*KS400 was inoculated (1% v/v) in 1 L of MRS broth. Batch fermentation was performed at 37 °C, without agitation, in the presence of nitrogen for 24 h. This procedure ensures an interruption of the bacterial growth prior to the stationary phase. Thus, it is prevented that proteases released into the extracellular medium degrade the bacteriocin.

### Preparation of bacteriocin extracts

Bacteriocin extracts were prepared according to the method described by Yang et al. (1992) with some modifications. Briefly, 1 L of culture medium from *La*KS400 cells fermentation was adjusted to pH 6.5 with 1 M NaOH solution followed by stirring at room temperature for 30 min, in order to allow the adsorption of bacteriocin to the producer cells. Then, the culture was heated at 70 °C for 30 min. Bacterial cells were harvested by benchtop centrifuge (Heraeus megafuge 8R centrifuge, Thermo-fisher Scientific, EUA) at 4500 rpm for 15 min at 4 °C and then were washed twice with 5 mM sodium phosphate (pH 6.5) and harvested by centrifugation in the same conditions. Then, bacterial cells were resuspended in 50 mL of 100 mM NaCl at pH 2.0 (adjusted with 5% phosphoric acid) and mixed with a magnetic stirrer for 1 h at 4 °C. The cell suspension was then centrifuged at 4500 rpm for 15 min at 4 °C and cell-free supernatant adjusted to pH 6.5 with 1 M NaOH solution. Bacteriocin extract obtained was filtrated through a 0.2 µm pore-size cellulose acetate (Fisher Scientific, UK) and the bioactivity was tested as previously described.

### Electrophoresis

As 1D protein electrophoresis (separation in one dimension) allows to separate biomolecules based on their size and structure, bacteriocin extract that exhibited bioactivity against the indicator strain was subjected to an electrophoresis in Tricine-SDS-PAGE on Bio-Rad Mini Protean 3 Cell apparatus (Bio-rad, USA). 4% polyacrylamide was used in the stacking gel and 16% polyacrylamide in the separation gel, as described by Schägger (2006). This procedure was made in duplicate. A 25 μL of bacteriocin sample was mixed with 25 μL of twofold concentrated buffer (Tris base 100 mM, glycine 100 mM, SDS 4%, urea 8 M, bromophenol blue 0.01%) and heated for 5 min at 100 °C. NZYColour Protein Marker II (NZYTech, Portugal), was used as molecular weight marker, with sizes ranging from 11 to 245 kDa. Electrophoresis was run at constant 35 mA for stacking gel and 50 mA for resolution gel.

In order to identify the position of the active bacteriocin, one half of the gel was fixed with methanol and acetic acid and washed three times with 250 mL of MiliQ Water for no longer than 30 min. The gel was then placed in a sterile petri dish and overlaid with MRS soft agar medium with 1% *Lactobacillus delbrueckii* ATCC 9649. The active bacteriocin was detected by macroscopic observation of an inhibition halo, after incubating at 37 °C, during 48 h in the presence of CO_2_ 10%. The other half of the gel was observed and photo-documented in Bio-Rad ChemiDoc MP in stain free mode. In addition, the influence of β-mercaptoethanol, on the bacteriocin antimicrobial activity was tested in order to confirm if the active protein had disulfide bridges. In fact β-mercaptoethanol is described as being responsible for the loss of the bacteriocin antimicrobial activity (Aslam et al. 2011; Deraz et al. 2007).

### Antimicrobial activity

The bacteriocin antimicrobial activity was assessed following the microdilution method as described elsewhere (Rex et al. 2008; Standards 2006). Briefly, wells from a microplate were filled with 100 μL of bacteriocin extract prepared as described previously and 100 μL of pathogen suspension in culture medium (containing 1 × 10^5^ cells assessed by optical density). Negative controls were performed corresponding to bacteriocin extract in sterile culture medium (2 wells for each type of culture medium used to support the pathogens growth); positive controls corresponded to 100 μL of pathogen suspension and 100 μL of PBS replacing the bacteriocin extract. The microplate was incubated at optimal growth conditions for the enrolled pathogens. After incubation, the presence or absence of pathogen growth in each well was compared to the growth obtained in the positive control. For this assay the enrolled pathogenic microorganisms were described on Table 1.

## Results

### Bacteriocin detection

**Fig. 1:**
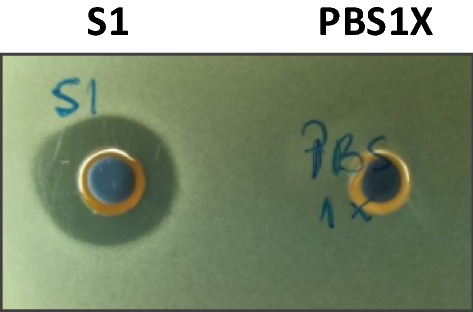
Antimicrobial activity of *Lactobacillus acidophilus* KS400 bacteriocin against *Lactobacillus delbrueckii* ATCC9649. Culture extracts of *Lactobacillus acidophilus* KS400 tested by the agar well diffusion method described (Tagg and McGiven 1971). S1—culture medium from *Lactobacillus acidophilus* KS400 medium; PBS 1X—negative control corresponding to ×1 phosphate buffered saline

The antimicrobial activity of the bacteriocin produced by LaKS400, obtained from the filtered culture medium after organic acids and hydrogen peroxide neutralization, showed a clear inhibition zone in agar when compared with negative control (PBS) (no inhibition observed), using *Lactobacillus delbrueckii* ATCC9649 as indicator organism (Fig. 1).

### Bacteriocin production and metabolites kinetic

**Fig. 2:**
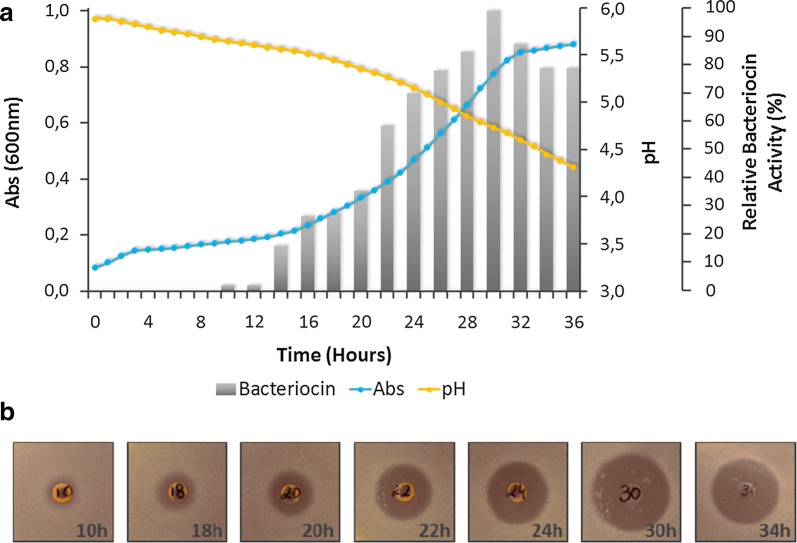
**a** Batch fermentation of *Lactobacillus acidophilus* KS400 strain in MRSB with non-controlled pH (initial value of 6.5) at 37 °C. Left axis: absorvance (Abs) at 600 nm; Primary right axis: pH values; Secondary right axis: Relative bacteriocin activity (%); Bottom axis: Time (hours). Symbols:  Optical density at 600 nm;  Changes in pH values; (-grey bars-) Bacteriocin activity production. **b** Antimicrobial activity of *Lactobacillus acidophilus* KS400 strain during fermentation; Activity was tested against *Lactobacillus delbrueckii* ATCC9649 and plates were incubated at 37 °C; 10% CO_2_

The organic acids produced by *Lactobacillus* (consequent to carbohydrates fermentation) induce a pH decrease of the medium. Batch-fermentation approach confirms a decrease in pH resulting from organic acid accumulation in the extracellular medium (Fig. 2, yellow line). After 36 h of LaKS400 fermentation the pH value was 4.3. In this study, the antimicrobial effect of organic acids was neutralized by the addition of a base solution, in order to eliminate its possible influence in the antimicrobial assessment of the bacteriocin. In addition, we have also considered that during batch fermentation included in our procedure, some amount of hydrogen peroxide could have been produced, thus we proceeded to its catalysation by adding catalase to the medium after the fermentation was completed. Thus, the relative bacteriocin activity was determined in relation to the sample that showed highest bioactivity (at time 30 h). The batch fermentation profiles of LaKS400 indicated that the bacteriocin production was more evident and clearly increased during the exponential growth phase, followed by a reduction during the stationary growth phase, as shown in Fig. 2a (grey bars).

After 32 h of fermentation a loss on the bacteriocin antimicrobial activity was observed (Fig. 2), by halo inhibition decrease.

### Bacteriocin molecular weight

**Fig. 3:**
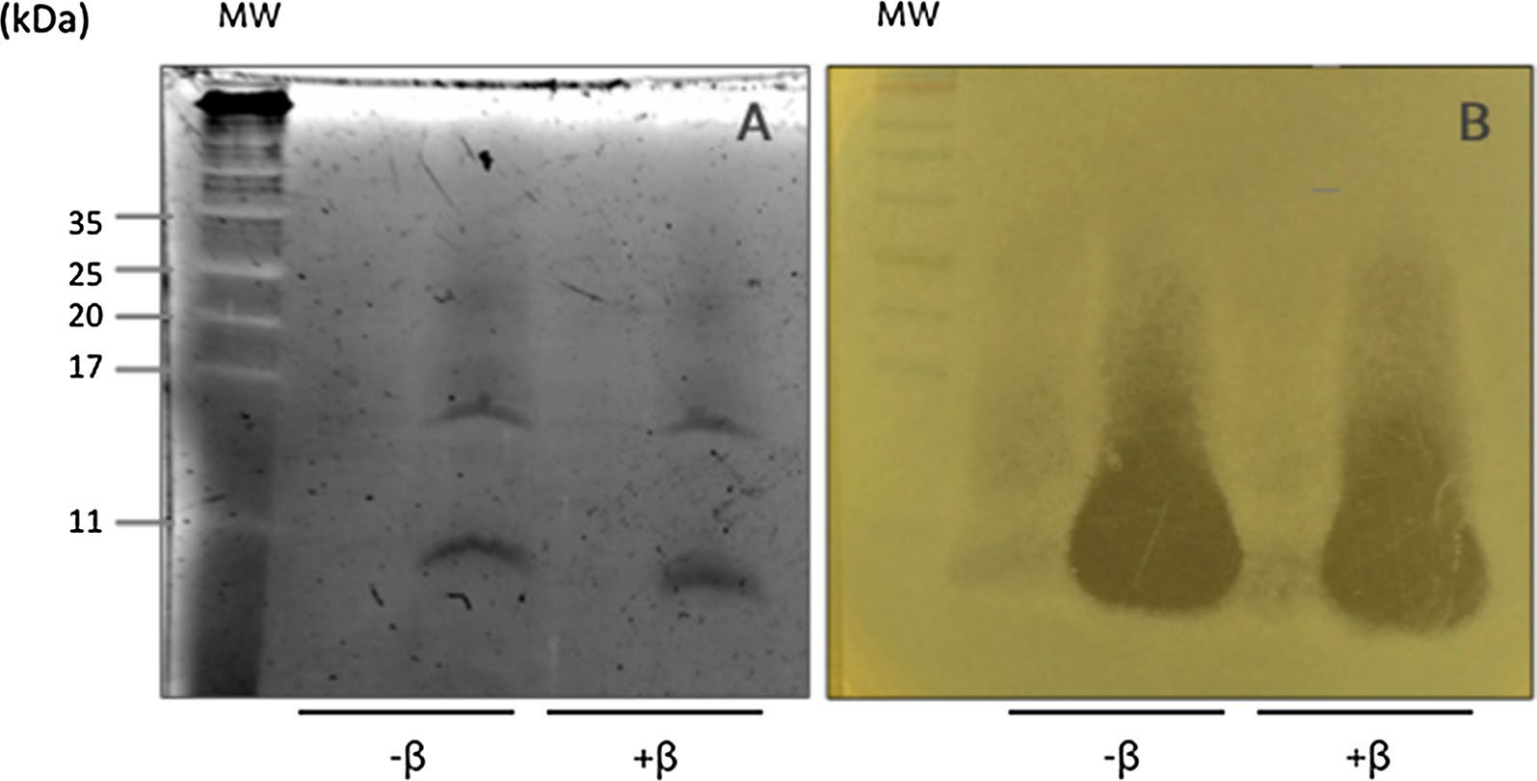
Direct detection of antimicrobial activity of *Lactobacillus acidophilus* KS400 bacteriocin (**A**) Tricine-SDS-PAGE gel was observed in ChemiDoc™ MP System (Biorad) using the stain free function for protein gels: (MW) molecular mass protein standards (NZYTECH, Portugal); − β/+ β—absence and presence of β-mercaptoethanol (**B**) Zone of growth inhibition corresponding to the position of the antimicrobial protein (bacteriocin)

Proteins were separated and a bioactivity assay was carried out with an indicator strain (*Lactobacillus delbrueckii* ATCC9649) directly on gel electrophoresis (Fig. 3b), in order to identify the active protein. Tricine-SDS-PAGE gel extraction of proteins resulted in a single zone of growth inhibition when the gel was overlaid with the indicator strain (Fig. 3). Adding β-mercaptoethanol did not influence the bacteriocin antimicrobial activity (+ β in Fig. 3).

The protein exhibiting antimicrobial activity in the electrophoresis gel corresponded to a band with an estimated molecular weight of approximately 7.5 kDa, considering that bioactivity and bacteriocin band was observed below to the 11 kD marker protein.

### Bacteriocin activity against pathogenic strains

The LaKS400 bacteriocin extract was tested against the indicator strain, pathogenic bacteria (including collection and clinical isolates) and *Candida albicans* ATCC10231 as shown in Table 2.

**Table 2:** Inhibitory spectrum of antimicrobial bacteriocin produced by *Lactobacillus acidophilus* KS400 against indicator strain and pathogenic microorganisms

Microorganisms	Source	Growth condition	Inhibition
*Lactobacillus delbrueckii* ATCC9649^a^	Collection	MRSB, 37 °C, 10% CO_2_	+
*Gardnerella vaginalis* 586876	Clinical	NYCIIIB, 37 °C, 10% CO_2_	+
*Gardnerella vaginalis* 563765	Clinical	NYCIIIB, 37 °C, 10% CO_2_	+
*Gardnerella vaginalis* 568799	Clinical	NYCIIIB, 37 °C, 10% CO_2_	+
*Staphylococcus aureus* ATCC6538	Collection	TSB, 37 °C, aerobic	−
*Streptococcus agalactiae* 181324	Clinical	BHIB, 37 °C, aerobic	+
*Streptococcus agalactiae* 179954	Clinical	BHIB, 37 °C, aerobic	+
*Pseudomonas aeruginosa* ATCC15442	Collection	TSB, 37 °C, aerobic	+
*Escherichia coli* ATCC8739	Collection	TSB, 37 °C, aerobic	−
*Candida albicans* ATCC10231	Collection	BHIB, 37 °C, aerobic	−

^a^Indicator microorganism

The bacteriocin extract was shown to inhibit the growth of the indicator strain *Lactobacillus delbrueckii* ATCC9649, *Gardnerella vaginalis*, *Streptococcus agalactiae* and *Pseudomonas aeruginosa*. No antimicrobial effect was observed against the enrolled strains of *Escherichia coli, Staphylococcus aureus* and *Candida albicans*.

## Discussion

This study aimed to evaluate the ability of LaKS400 to produce bacteriocin with antimicrobial activity against urogenital pathogens, expressing mechanism of action that was not previously reported in other in vitro studies performed for this strain (Ünlü and Donders 2011).

The bacteriocin production and detection was performed in vitro and accompanied by the production of other bacterial metabolites (such as lactic acid and hydrogen peroxide) that were previously described for this strain. Thus, to assure that the bioactivity assessed for the protein extract was not related to these metabolites, their effect was neutralized. Thus, the experimental results suggest that a proteinaceous antimicrobial compound, bacteriocin, had been produced and secreted by LaKS400 into the extracellular culture medium. The bacteriocin production occurs during the exponential phase reaching its maximum during the stationary phase of the strain growth curve (between 24 and 30 h after the start of fermentation), as described for other bacteriocins (De Vuyst et al. 1996; De Vuyst and Vandamme 1992; Zamfir et al. 2000). However, after 32 h of fermentation a loss on the bacteriocin antimicrobial activity was observed. This change in the bioactivity can be related to protein aggregation or proteolytic degradation of the bacteriocin (Law and Kolstad 1983).

Based on the described results we conclude that LaKS400 produces a bacteriocin with an expected size of 7.5 kDa. This molecular weight is within the range of the most frequently reported bacteriocins from *Lactobacillus* spp. (Vuyst and Vandamme 1994). In fact, for *Lactobacillus acidophilus*, the size described for bacteriocins ranges from 2.5 to 100 kda (Barefoot and Klaenhammer 1983; Muriana and Klaenhammer 1991; Zamfir et al. 1999). In addition, no decrease on antimicrobial activity was observed for bacteriocin extract in the presence of β-mercaptoethanol. This result suggests that the activity was not related to the presence of disulfide bridges. These are characteristic of class II bacteriocins, since its contains at least two cysteines in their composition that forms a bond (Ennahar et al. 2000). Disulfide bridges have been described in some *Lactobacillus* spp., such as *L. curvatus* (Tichaczek et al. 1993), *L. plantarum* (Ennahar et al. 1996) and *L. sake* (Schillinger and Lücke 1989), among others. Further studies may be carried out that include cloning, expression and isolation in vector and host microorganism in order to elucidate about its function.

In parallel, we conducted antimicrobial tests to access the bacteriocin extract bioactivity against several urogenital pathogens. Bacteriocin extract was shown to inhibit the growth of the indicator strain, Gram-positive (*Gardnerella vaginalis* and *Streptococcus agalactiae*) and Gram-negative bacteria *Pseudomonas aeruginosa*. However, no antimicrobial effect was observed against the enrolled strains of *Escherichia coli, Staphylococcus aureus* and *Candida albicans*. This finding was not unexpected as bacteriocins act mainly against the closely related species, while against fungi, the lactobacilli activity, if at all present, is probably related with different antimicrobial effects, like competition for adhesion sites. Similar results were observed in another published study (Mitra et al. 2005), while it has been described that bacteriocins produced by Lactic acid bacteria (including *Lactobacillus*) are primarily active against Gram-positive bacteria (Heng et al. 2007; Jack et al. 1995). Moreover, several studies have been published regarding bacteriocins produced by *Lactobacillus acidophilus*. For example, *Lactobacillus acidophilus* DSM20079 produced an antimicrobial bacteriocin (acidocin D20079) with approximately 6.6 kDa that had an inhibitory activity against *Lactobacillus* genus, including *Lactobacillus delbrueckii* subsp. *lactis* DSM20076, *Lactobacillus bulgaricus* DSM20080 and *Lactobacillus sakei* NCDO2714, but no evident activity was observed against other pathogens (Deraz et al. 2005). Furthermore, it has been also described by other authors that active bacteriocins produced by *Lactobacillus acidophilus*, are small peptides with different physicochemical properties. In fact, for *Lactobacillus acidophilus* JCM1132 a heat-stable two-component bacteriocin (J1132) exhibiting a narrow inhibitory activity against non-pathogenic strains has been described (Tahara et al. 1996), and a bacteriocin with 3.1 kDa active against pathogenic strains was identified for *Lactobacillus acidophilus* ATCC4356 (Han et al. 2002).

These results corroborate the LaKS400 probiotic efficacy in vaginal application or its use in other pathologies. In fact, this study reports for the first time a new bacteriocin produced by LaKS400, with an expected size of 7.5 kDa and antimicrobial activity against vaginal pathogen microorganisms, specifically *Gardnerella vaginalis* and *Streptococcus agalactiae*, two most relevant pathogens related to vaginal infections. These results corroborate a new mechanism of action for this probiotic strain and support its use in clinical practice for the treatment and prevention of vaginal infections. Further studies may be carried out to improve bacteriocin production, isolation, purification and elucidate about its possible mechanism of action.

## Abbreviations

LaKS400: *Lactobacillus acidophilus* KS400
MRS: De Man, Rogosa and Sharpe
PBS: phosphate-buffered saline
